# Histological Structure and Immunohistochemical Properties of the Ligamentum Teres in Patients With Developmental Dysplasia of the Hip

**DOI:** 10.7759/cureus.59748

**Published:** 2024-05-06

**Authors:** Baran Sarıkaya, Mehmet Ali Dolap, Ahmet Yiğit Kaptan, Celal Bozkurt, Nihat Yumuşak, Akin Yigin, Serkan Sipahioğlu, Baki Volkan Çetin, Mehmet Akif Altay

**Affiliations:** 1 Department of Orthopaedics and Traumatology, University of Health Sciences, Ankara Bilkent City Hospital, Ankara, TUR; 2 Department of Orthopaedics and Traumatology, Faculty of Medicine, Harran University, Sanliurfa, TUR; 3 Department of Orthopaedics and Traumatology, Gaziosmanpaşa Taksim Training and Research Hospital, University of Health Sciences, Istanbul, TUR; 4 Department of Pathology, Faculty of Veterinary, Harran University, Sanliurfa, TUR; 5 Department of Genetics, Faculty of Veterinary, Harran University, Sanliurfa, TUR; 6 Department of Orthopaedics and Traumatology, Faculty of Medicine, Ordu University, Ordu, TUR

**Keywords:** adamts, metalloproteinases, deformity, ligamentum teres, hip dypslasia

## Abstract

Introduction

This study aims to evaluate the histology of the ligamentum teres and its relationship with matrix metalloproteinases (MMPs) and a disintegrin and metalloproteinase with thrombospondin motifs (ADAMTS), which are involved in the destruction of extracellular matrix proteins in patients with developmental dysplasia of the hip (DDH).

Methodology

The patients who underwent open reduction and pelvic osteotomy due to DDH were included in the study. Patient groups were formed according to Tönnis stages, positive family history, consanguineous marriage, age, and bilateral involvement. The histology and immunohistochemical properties (MMP-2, MMP-9, and ADAMTS-7) of ligamentum teres tissue obtained from the patients were evaluated according to these groups.

Results

Thirty-five patients (female 30, 85.7%; male 5, 14.3%) with DDH between the ages of 14 and 99 months were included in the study. Preoperative and postoperative Tönnis stages, positive family history, consanguineous marriage, age, and bilaterality did not cause a significant difference between histological parameters. A significant correlation was found between MMP-2, MMP-9, and ADAMTS-7 and all histological parameters.

Conclusions

The histological structure of ligamentum teres in patients with DDH shows moderate inflammation, fibrosis, neovascularization, hyalinization, and fatty infiltration regardless of age and radiological stage. ADAMTS-7, MMP-2, and MMP-9 correlate positively with the histological parameters of the ligamentum teres in patients with DDH.

## Introduction

The ligamentum teres has been defined as a broad-based, triangular, and slightly flattened ligament with an overlying layer of investing synovium, and it was demonstrated that the part of the ligament between the femoral head and the acetabular margin had the appearance and the consistency of cartilage [1-3]. However, in developmental dysplasia of the hip (DDH), due to the change in the hip anatomy, the ligamentum teres becomes elongated and hypertrophied in accordance with the capsule [1]. Therefore, it is considered one of the potential obstacles to reduction in DDH.

There are many studies in the literature regarding the anatomy and biomechanics of the ligamentum teres [1-10]. With the demonstration of the effect of the ligament teres on hip stabilization, some authors suggested preserving the ligamentum teres during open reduction in patients with DDH [3,4,10]. However, the histological structure of the ligament was not investigated in these studies, and there are a limited number of studies regarding the histology of the ligamentum teres in patients with DDH [1].

Matrix metalloproteinases (MMPs) and a disintegrin and metalloproteinase with thrombospondin motifs (ADAMTS) enzymes are proteases that are involved in the destruction of extracellular matrix (ECM) proteins [11,12]. ADAMTS-7 gene from the ADAMTS family is expressed in muscle, bone, ligament, meniscus, skeletal muscle, fat tissue, and cartilage tissues containing cartilage oligometric matrix protein (COMP) [13]. COMP is one of the ECM proteins and is a well-known substrate of active ADAMTS-7. Current studies emphasize the role of ADAMTS-7 in the regulation of collagen, which forms the ligament structure [13]. In addition, MMP-2 (gelatinase A) and MMP-9 (gelatinase B), which are members of the MMP family, have been reported to have crucial roles in collagen degradation and tendon physiology [14].

This study aims to analyze the histological structure of the ligamentum teres in patients with DDH and put forth its relationship with ECM proteases, which have critical functions in protecting the ligament structure. We also aimed to examine the relationship between the demographic and radiological features of DDH with the histological and immunohistochemical structure of the ligamentum teres.

## Materials and methods

This research was approved by the IRB of the authors’ affiliated institutions (HRU/20.02.25). The study was conducted at Harran University Faculty of Medicine Hospital. Informed consent was obtained from each patient’s parents. The patients who underwent open reduction and pelvic osteotomy due to DDH between May and December 2020 were included in the study. The ligamentum teres excised during surgery was evaluated histologically, and Tönnis staging was used for the radiological classification of all patients both before surgery and after cast termination. Patients with neuromuscular and genetic disorders associated with teratological hip dislocation were excluded from the study. A total of 35 patients (female 30, 85.7%; male 5, 14.3%) with DDH aged between 14 and 99 months were included in the study. Demographics of the patients are given in Table 1.

**Table 1:** Demographics of the patients.

Demographic variables		Count, *n*	%
Gender	Female	30	85.7
	Male	5	14.3
Age (month)	<24	17	48.6
	>24	18	51.4
Preop Tönnis grade	1-2	7	20.0
	3-4	28	80.0
Post-op Tönnis grade	1.00	30	85.7
	2.00	2	5.7
	3.00	3	8.6
Bilateral	Yes	21	60.0
	No	14	40.0
Family history	Yes	6	17.1
	No	29	82.9
Consanguineous marriage	Yes	19	54.3
	No	16	45.7

Immunohistochemistry

For immunohistochemical studies, 4-µm-thick sections were obtained from the paraffin-embedded tissue blocks on poly-L-lysine-coated glass slides. They were stained with the streptavidin-biotin-peroxidase complex (ABC) technique after routine deparaffinization and rehydration procedures (Zymed, Histostain Plus Kit, CA). Antigen retrieval was performed in a microwave oven with citrate buffer (pH 6.0; 700 W, 20 minutes). Endogenous peroxidase activation in the tissues was blocked for 15 minutes with 0.3% hydrogen peroxide in 0.01 M phosphate-buffered saline (PBS) in methanol at room temperature. Before applying the primary antibody, the tissues were incubated for 20 minutes with 5% normal goat serum for protein blocking. Then sections were incubated with MMP-2 (1:100, Invitrogen, CA-4001), MMP-9 (1:50, Invitrogen, PA5-13199), and ADAMTS-7 (1:100, Abcam, ab28557) primer antibodies for one hour at room temperature. Sections were then reacted with a biotinylated secondary antibody for 30 minutes after removing the unbound primary antibody. Then, the sections were reacted with horseradish peroxidase (HRP)-streptavidin for 30 minutes. The diaminobenzidine (DAB, Dako, Glostrup, Denmark) for MMP-9 and AEC (3-Amino 9-Ethyl Carbasole, Dako) for ADAMTS-7 and MMP-2 was used as the chromogen. Finally, the background of the tissue sections was stained with hematoxylin. All staining steps were carried out at 37 °C and in humidity cabinets. PBS solution was used as a wash-away solution during all the staining steps. To assess the immunohistochemistry, 10 fields were randomly chosen and the cytoplasmic staining intensity in the cells was globally scored: negative, 0 (<1% positive); weak, 1 (1%-25% positive); intermediate, 2 (>25% to 75% positive); and strong, 3 (>75% positive).

Histopathological studies

To remove the fixed tissue samples from formalin, they were washed in running water overnight. Afterward, it was subjected to routine pathological tissue follow-up and passed through graded alcohol (50%, 75%, 96%, and 100%) and xylol series and blocked in paraffin. Five-micrometer-thick sections were separated from the prepared blocks and the first three sections, and every tenth section was taken on slides with Leica RM 2125 RT. The prepared preparations were passed through alcohol and xylol series and stained with hematoxylin and eosin (H&E). All samples were examined under a high-resolution light microscope (Olympus DP-73 camera, Olympus BX53-DIC microscope, Tokyo, Japan). All the changes detected in tissue structures were noted and graded according to the presence and severity of any particular finding as 0, none; 1, mild (<25%); 2, moderate (25%-50%); and 3, severe (>50%).

Statistical analyses

The normality of the distribution of continuous variables was tested by the Shapiro-Wilk test. Kruskal-Wallis and Dunn multiple comparison tests were used to compare non-normal numerical data among groups. Spearman rank correlation coefficients were calculated for correlations between non-normal numerical variables. Mean ± standard deviations (mean ± SD) and median and interquartile ranges were given as descriptive statistics. Statistical analysis was performed with IBM SPSS Statistics for Windows, Version 24.0 (IBM Corp., Armonk, NY), and a *P*-value < 0.05 was accepted as statistically significant.

## Results

Four open reduction and 31 open reduction + pelvic osteotomies were performed as surgical procedures. The mean age of the patients was 27.71 ± 16.6 months, and the follow-up duration was 7.89 ± 3.8 months. Additional histological data are given in Table 2.

**Table 2:** Treatment results and histological data of the patients. Significant at 0.05 level. Mann-Whitney U test. Inf, inflammation; Fib, fibroblast; Neo, neovascularization; Sinf, synovial inflammation; Hyl, hyalinization; Shyp, synovial lining cell hyperplasia; Cclu, condrocyte clustering; Fatinf, fat infiltration; IMMP-2, immunohistochemical analysis of MMP-2; IMMP-9, immunohistochemical analysis of MMP-9; IADAMTS-7, immunhistochemical analysis of ADAMTS-7

Variables	Median (min-max)
Preop Tönnis grade	3 (1-4)
Postop Tönnis grade	1 (1-3)
IMMP-2	2 (0-2)
IMMP-9	2 (0-3)
IADAMTS-7	2 (0-3)
Inf	2 (0-2)
Fib	2 (0-3)
Neo	2 (0-3)
Sinf	2 (0-3)
Hyl	2 (0-3)
Shyp	2 (1-3)
Cclu	2 (1-3)
Fatinf	2 (0-3)

Preoperative and postoperative Tönnis stages had no significant effects on histological parameters (Table 3). The presence of DDH in the family history, consanguineous marriage, and bilaterality did not cause a significant difference between histological parameters (Table 4). In addition, when the patients were divided into two groups younger than 24 months old and older, no significant difference was found between the histological parameters between the two groups (Table 4).

**Table 3:** Comparison of histological data according to pre-op Tönnis staging and post-op Tönnis staging. *Significant at 0.05 level. Mann-Whitney U test. **Significant at 0.05 level. Kruskal-Wallis test. Inf, inflammation; Fib, fibroblast; Neo, neovascularization; Sinf, synovial inflammation; Hyl, hyalinization; Shyp, synovial lining cell hyperplasia; Cclu, condrocyte clustering; Fatinf, fat infiltration; IMMP-2, immunohistochemical analysis of MMP-2; IMMP-9, immunohistochemical analysis of MMP-9; IADAMTS-7, immunhistochemical analysis of ADAMTS-7

	Pre-op Tönnis 1-2 (*n* = 7), Median (25%-75%)	Pre-op Tönnis 3-4 (*n* = 28), Median (25%-75%)	*P*-value*	Post-op Tönnis 1 (*n* = 30), Median (25%-75%)	Post-op Tönnis 2 (*n* = 2), Median (25%-75%)	Post-op Tönnis 3 (*n* = 3), Median (25%-75%)	*P*-value**
Inf	1 (0-2)	2 (1-2)	0.146	2 (1-2)	1 (0-2)	1 (0-2)	0.686
Fib	1 (1-3)	2 (1.5-2)	0.643	2 (1-2)	2 (2-2)	2 (1-2)	0.898
Neo	2 (2-3)	2 (1.5-2.5)	0.672	2 (2-3)	2 (2-2)	2 (0-2)	0.505
Sinf	0 (0-2)	2 (0-2)	0.146	2 (0-2)	1 (0-2)	0 (0-2)	0.521
Hyl	2 (2-2)	2 (1.5-2)	0.920	2 (2-2)	2 (2-2)	2 (0-2)	0.622
Shyp	2 (2-3)	2 (1.5-3)	0.762	2 (2-3)	2.5 (2-3)	2 (1-3)	0.674
Cclu	2 (2-3)	2 (1.5-3)	0.856	2 (2-3)	2.5 (2-3)	2 (1-2)	0.444
Fatinf	2 (2-3)	2 (1-2.5)	0.558	2 (1-3)	2 (2-2)	2 (0-3)	0.982
IMMP2	2 (0-2)	2 (1-2)	0.672	2 (1-2)	2 (2-2)	2 (0-2)	0.561
IMMP9	2 (1-2)	2 (2-3)	0.340	2 (2-3)	2.5 (2-3)	2 (1-2)	0.459
IADAMTS7	2 (1-3)	2 (2-3)	0.479	2 (2-3)	2.5 (2-3)	2 (1-2)	0.425

**Table 4:** Comparison of histological data according to family history, consanguineous marriage, age of patients, and bilateral hip involvement. *Significant at 0.05 level. Mann-Whitney U test. Inf, inflammation; Fib, fibroblast; Neo, neovascularization; Sinf, synovial inflammation; Hyl, hyalinization; Shyp, synovial lining cell hyperplasia; Cclu, condrocyte clustering; Fatinf, fat infiltration; IMMP-2, immunohistochemical analysis of MMP-2; IMMP-9, immunohistochemical analysis of MMP-9; IADAMTS-7, immunhistochemical analysis of ADAMTS-7

Histological variables	Family history + (*n* = 6), median (25%-75%)	Family history – (*n* = 29), median (25%-75%)	*P*-value*	Consanguineous marriage + (*n* = 19), median (25%-75%)	Consanguineous marriage – (*n* = 16), median (25%-75%)	*P*-value*	Age < 24 months (*n* = 17), median (25%-75%)	Age > 24 months (*n* = 18), median (25%-75%)	*P*-value*	Bilateral + (*n* = 21), median (25%-75%)	Bilateral – (*n* = 14), median (25%-75%)	*P*-value*
Inf	1.5 (0-2)	2 (1-2)	0.681	1 (0-2)	2 (1-2)	0.103	1 (0-2)	2 (1-2)	0.130	2 (1-2)	1.5 (0-2)	0.402
Fib	1.5 (1-2)	2 (1-2)	0.492	2 (1-2)	2 (1.5-2.5)	0.298	2 (1-2)	2 (2-2)	0.158	2 (2-2)	2 (1-2)	0.130
Neo	2 (1-3)	2 (2-2)	0.962	2 (1-2)	2 (2-3)	0.427	2 (1-2)	2 (2-3)	0.272	2 (2-3)	2 (1-2)	0.270
Sinf	2 (1-2)	2 (0-2)	0.541	1 (0-2)	2 (0.5-2)	0.171	2 (0-2)	2 (0-2)	0.883	2 (0-2)	2 (0-2)	0.865
Hyl	2 (1-2)	2 (2-2)	0.709	2 (1-2)	2 (2-2.5)	0.337	2 (1-2)	2 (2-2)	0.302	2 (2-2)	2 (1-2)	0.515
Shyp	2 (1-3)	2 (2-3)	0.849	2 (1-3)	2 (2-3)	0.615	2 (1-3)	2 (2-3)	0.668	2 (2-3)	2 (1-2)	0.391
Cclu	2 (1-2)	2 (2-3)	0.358	2 (1-2)	2 (2-3)	0.219	2 (1-2)	2 (2-3)	0.110	2 (2-3)	2 (1-2)	0.301
Fatinf	2 (1-3)	2 (2-2)	0.944	2 (1-3)	2 (2-2.5)	0.383	2 (2-2)	2 (1-3)	0.958	2 (1-3)	2 (2-2)	0.677
IMMP2	1.5 (1-2)	2 (1-2)	0.819	1 (1-2)	2 (2-2)	0.067	2 (1-2)	2 (1-2)	0.514	2 (1-2)	2 (1-2)	0.969
IMMP9	2 (1-3)	2 (2-3)	0.814	2 (1-2)	2 (2-3)	0.122	2 (2-3)	2 (2-3)	0.845	2 (2-2)	2 (2-3)	0.437
IADAMTS7	2 (1-3)	2 (2-3)	0.606	2 (1-3)	2.5 (2-3)	0.271	2 (2-3)	2 (2-3)	0.646	2 (2-3)	2 (2-3)	0.928

The correlation between the immunohistochemical analysis of MMP-2 (IMMP-2), immunohistochemical analysis of MMP-9 (IMMP-9), immunohistochemical analysis of ADAMTS-7 (IADAMTS-7), and histological data was evaluated. A significant correlation was found between IMMP-2, IMMP-9, and IADAMTS-7 and all histological parameters. A moderate correlation was found for the R-value between 0.4 and 0.6, a strong correlation for 0.6 and 0.8, and a very strong correlation for a value of 0.8 and above (Table 5).

**Table 5:** Comparison of histological data according to IMMP-2, IMMP-9, and IADAMTS-7. *Significant at 0.01 level. *r*, Spearman rank correlation coefficient; Inf, inflammation; Fib, fibroblast; Neo, neovascularization; Sinf, synovial inflammation; Hyl, hyalinization; Shyp, synovial lining cell hyperplasia; Cclu, condrocyte clustering; Fatinf, fat infiltration; IMMP-2, immunohistochemical analysis of MMP-2; IMMP-9, immunohistochemical analysis of MMP-9; IADAMTS-7, immunhistochemical analysis of ADAMTS-7

Histological variables		IMMP-2	IMMP-9	IADAMTS-7
Inf	r	0.515^*^	0.592^*^	0.580^*^
	P	0.002	0.001	0.001
Fib	r	0.757^*^	0.720^*^	0.775^*^
	P	0.001	0.001	0.001
Neo	r	0.730^*^	0.772^*^	0.813^*^
	P	0.001	0.001	0.001
Sinf	r	0.465^*^	0.709^*^	0.682^*^
	P	0.005	0.001	0.001
Hyl	r	0.720^*^	0.757^*^	0.755^*^
	P	0.001	0.001	0.001
Shyp	r	0.736^*^	0.803^*^	0.776^*^
	P	0.001	0.001	0.001
Cclu	r	0.745^*^	0.717^*^	0.741^*^
	P	0.001	0.001	0.001
Fatinf	r	0.625^*^	0.661^*^	0.591^*^
	P	0.001	0.001	0.001

Histological results

**Figure 1:**
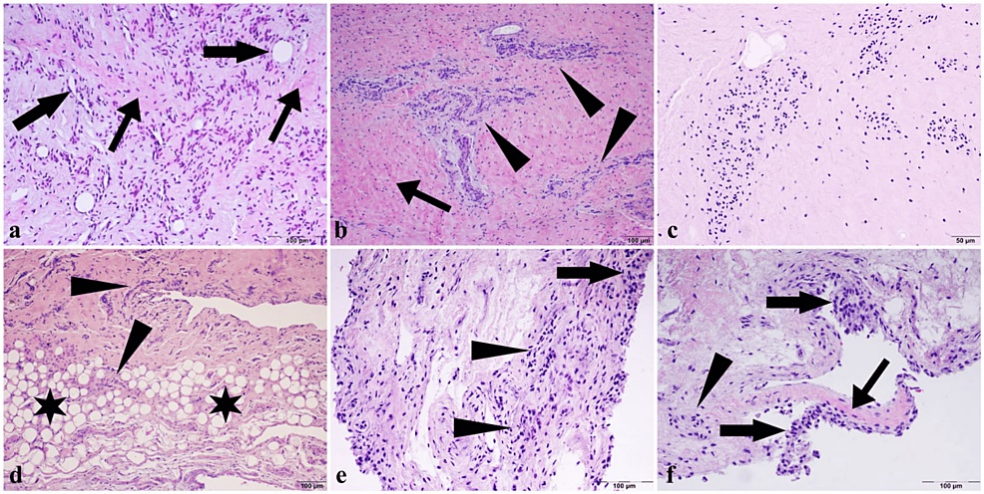
Histomorphological examination of the tissues. (a) Hyalinized fibrous tissue increase (thin arrows) and numerous new vascular formations (thick arrows) in the damaged ligament with inflammatory cells. (b) Hyalinization (arrow) and severe inflammation (arrowheads) of cartilage tissue in large areas. (c) Chondrocytes clustering into focal areas. (d) Diffuse inflammation (arrowheads) with fat infiltration (stars) over large areas of the synovial structure. (e) Areas of hyperplasia (arrow) and severe inflammation (arrowheads) in synovial membrane cells. (f) Hyalinization (thin arrow) and focal inflammation (arrowhead) with cellular hyperplasia (thick arrows) of the synovial membrane (H&E staining, bar: 100 µm). H&E, hematoxylin and eosin

Histomorphological examination of the tissues revealed varying severity of damage. Inflammation (Figures 1a-1b) and clustering of chondrocytic cells (Figure 1c) were observed focally in some areas. It was also observed that fibrosis was formed around the inflammation and hyalinization. Fat infiltrates spreading as focal areas in the ligamentum teres and synovial membrane (Figure 1d) were noted, along with an increase in neovascular structures (Figure 1a). Similar damage and inflammation also occurred in the synovial membrane, and cellular hyperplasia was also noted (Figures 1e-1f).

Immunohistochemical results

**Figure 2:**
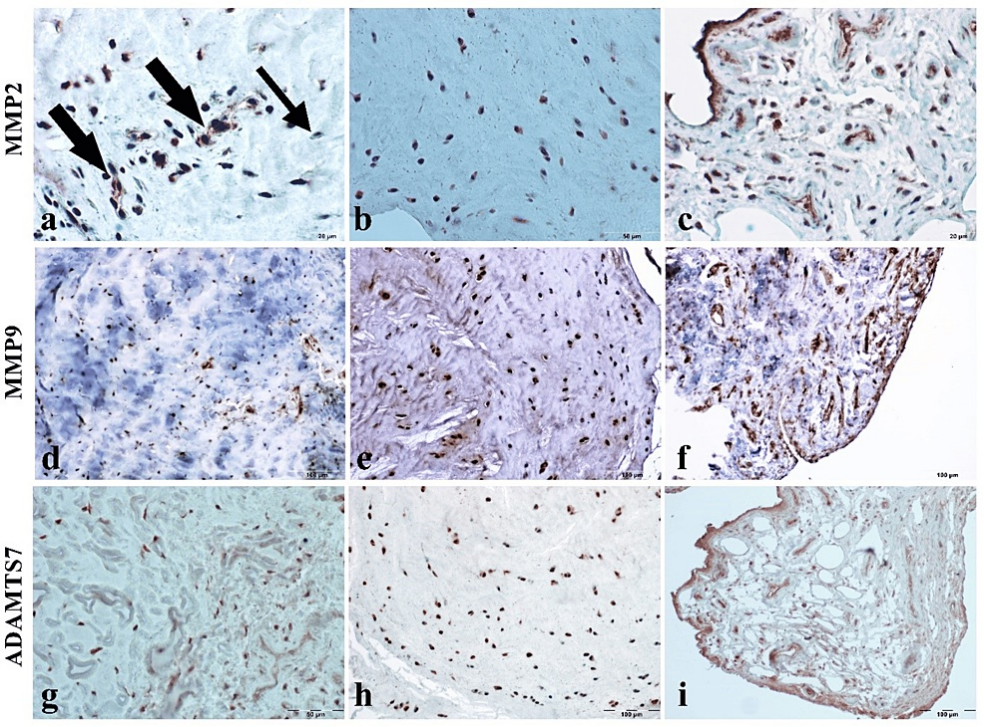
Immunohistochemical-positive reactions with MMP-2, MMP-9, and ADAMTS-7 antibodies. Immunohistochemical staining findings. (a) MMP-2 severe cytoplasmic positive cells (thick arrows) and occasional negative reaction (thin arrow; bar 20 µm). (b) Diffuse MMP-2-positive chondrocytic cells (bar 50 µm). (c) Intense positive reaction in the synovial membrane (bar 20 µm). (d) and (e) Dense and severe MMP-9 immunopositive cells in the ligament. (f) Positive cell density in synovial membrane cells. (g) and (h) Dense and severely immunopositive cells in the ligament for the ADAMTS-7 antibody. (i) ADAMTS-7-positive cell density in the synovial membrane (ABC staining). ABC, Avidin-Biotin Complex

It was determined that there were positive reactions with varying intensities in the studies performed in terms of MMP-2, MMP-9, and ADAMTS-7 antibodies immunohistochemically (Figure 2). In examinations conducted for MMP-2, intense positive reactions were observed in both chondrocytes and the synovial membrane. However, the presence of negative cells was noted in some areas. On the other hand, the results of the study conducted with MMP-9 and ADAMTS-7 antibodies revealed a widespread positive reaction in both the ligamentum teres and the synovia.

## Discussion

This study shows that the histological structure of the ligamentum teres and IMMP-2, IMMP-9, and IADAMTS-7 in patients with DDH is not affected by age, stage of dysplasia, family history, bilateral dysplasia, and consanguineous marriage. IMMP-2, IMMP-9, and IADAMTS-7 showed a positive correlation with ligamentum teres histological features.

It was shown that degenerative and inflammatory processes in tissues increase as a result of ECM degradation when ADAMTS-7, MMP-2, and MMP-9 were overexpressed [13,15]. The increase of these proteases in an injured tendon can be an indicator of tissue remodeling, repair, and healing [12-17]. As it is seen, these metalloproteinases can be involved in the pathogenesis of degenerative and inflammatory diseases, as well as in the repair and remodeling processes of healing tissues. ADAMTS-7 gene, together with MMP-2 and MMP-9 enzymes, play an important role in ECM regulation in tendon physiology [12-14]. Karousou et al. [14] compared MMP activity in ruptured and healthy parts of the Achilles tendon and showed that MMP-2 and MMP-9 activity was increased in the ruptured tendon region. Mead et al. [17] showed the effect of ADAMTS-7 on tendon organization in their study on mice. Accordingly, in our study, we found that deterioration in histological parameters correlated positively with IMMP-2, IMMP-9, and IADAMTS-7. This result shows that the ADAMTS-7 gene has an effect on ligament healing together with MMP-2 and MMP-9 enzymes per the literature.

In DDH disease, the histological structure of the ligamentum teres differentiates as it elongates and becomes hypertrophied. Ipplito et al. [1] studied the histological structure of the ligamentum teres in patients with DDH and reported that ligamentum teres had fibrocartilaginous metaplasia close to the femoral insertion. In addition, they added that the arteriolar walls were thicker in patients with DDH and that the thickness increased with age. Similar to Ippliton et al.'s findings, we also found moderate fibrosis, hyalinization, and chondrocyte clustering in the ligamentum teres of patients with DDH. Differently, we found moderate neovascularization and fatty infiltration in this study. In addition, there were nine patients in the study of Ipplito et al., while there were 35 patients in our study. According to the results of the present study, pediatric orthopedics who prefer tenodesis by shortening and suturing this ligament during DDH open reduction surgery should also take into consideration the altered structure of the ligament.

Some recent studies suggested shortening and reattaching the ligamentum teres into the acetabulum instead of excision in DDH surgery. Wenger et al. showed the biomechanical effect of the ligamentum teres with an in vitro porcine model [3] and later reported early positive results with the ligamentum teres reattachment technique in patients with DDH [10]. Bache et al. [4] also described the ligamentum teres tenodesis technique with medial open reduction and reported good results in patients up to two years of age in their study. Contrary to these studies, Ertürk et al. [18] reported that there are unmyelinated free nerve endings in the ligamentum teres structure, and since these receptors play a role in pain sensation, ligamentum teres excision does not cause a significant loss of sensory function. Although the methods and necessity of ligamentum teres tenodesis are not the subjects of this study, the present study reveals that the age and radiological stage of DDH do not have a significant effect on ligamentum teres structure.

The findings of this study have to be seen in light of some limitations. First, there was a limited number of patients included in the study. Second, histological comparison could not be made with healthy ligamentum teres tissue because only patients with DDH were included in the study. In addition, genetic analysis of ADAMTS-7, MMP-2, and MMP-9 was not evaluated.

## Conclusions

The histopathological studies on the ligamentum teres are quite insufficient in the literature. The limited number of studies includes mostly adults in the adult age group. We could not find a recent study examining the histological or histopathological features of the ligamentum teres in children with DDH. Therefore, the findings we obtained in this study may contribute to future histopathological studies.

In conclusion, the histological structure of the ligamentum teres in patients with DDH shows moderate inflammation, fibrosis, neovascularization, hyalinization, and fatty infiltration regardless of age and radiological stage. ADAMTS-7, MMP-2, and MMP-9 correlate positively with the histological parameters of the ligamentum teres in patients with DDH.

## References

[1] Ipplito E, Ishii Y, Ponseti IV (1980). Histologic, histochemical, and ultrastructural studies of the hip joint capsule and ligamentum teres in congenital dislocation of the hip. Clin Orthop Relat Res.

[2] Kaku N, Shimada T, Tabata T, Tagomori H, Abe T, Zhang JJ, Tsumura H (2017). Three-dimensional architecture of the ligamentum teres in the human hip joint. Muscles Ligaments Tendons J.

[3] Wenger D, Miyanji F, Mahar A, Oka R (2007). The mechanical properties of the ligamentum teres: a pilot study to assess its potential for improving stability in children's hip surgery. J Pediatr Orthop.

[4] Bache CE, Graham HK, Dickens DR (2008). Ligamentum teres tenodesis in medial approach open reduction for developmental dislocation of the hip. J Pediatr Orthop.

[5] Catto M (1965). A histological study of avascular necrosis of the femoral head after transcervical fracture. J Bone Joint Surg Br.

[6] Dehao BW, Bing TK, Young JL (2015). Understanding the ligamentum teres of the hip: a histological study. Acta Ortop Bras.

[7] Jo S, Hooke AW, An KN, Trousdale RT, Sierra RJ (2018). Contribution of the ligamentum teres to hip stability in the presence of an intact capsule: a cadaveric study. Arthroscopy.

[8] Kivlan BR, Richard Clemente F, Martin RL, Martin HD (2013). Function of the ligamentum teres during multi-planar movement of the hip joint. Knee Surg Sports Traumatol Arthrosc.

[9] Martin HD, Hatem MA, Kivlan BR, Martin RL (2014). Function of the ligamentum teres in limiting hip rotation: a cadaveric study. Arthroscopy.

[10] Wenger DR, Mubarak SJ, Henderson PC, Miyanji F (2008). Ligamentum teres maintenance and transfer as a stabilizer in open reduction for pediatric hip dislocation: surgical technique and early clinical results. J Child Orthop.

[11] Haberal B, Şimşek EK, Baysan Çebi HP, Tuç Ö, Verdi H, Ataç FB (2021). Lack of association between MMP13 (rs3819089), ADAM12 (rs3740199-rs1871054) and ADAMTS14 (rs4747096) genotypes and advanced-stage knee osteoarthritis. Jt Dis Relat Surg.

[12] Malemud CJ (2019). Inhibition of MMPs and ADAM/ADAMTS. Biochem Pharmacol.

[13] Zhang Y, Lin J, Wei F (2015). The function and roles of ADAMTS-7 in inflammatory diseases. Mediators Inflamm.

[14] Karousou E, Ronga M, Vigetti D, Passi A, Maffulli N (2008). Collagens, proteoglycans, MMP-2, MMP-9 and TIMPs in human achilles tendon rupture. Clin Orthop Relat Res.

[15] Lipari L, Gerbino A (2013). Expression of gelatinases (MMP-2, MMP-9) in human articular cartilage. Int J Immunopathol Pharmacol.

[16] Del Buono A, Oliva F, Osti L, Maffulli N (2013). Metalloproteases and tendinopathy. Muscles Ligaments Tendons J.

[17] Mead TJ, McCulloch DR, Ho JC, Du Y, Adams SM, Birk DE, Apte SS (2018). The metalloproteinase-proteoglycans ADAMTS7 and ADAMTS12 provide an innate, tendon-specific protective mechanism against heterotopic ossification. JCI Insight.

[18] Ertürk C, Koçarslan S, Büyükdoğan H, Altay MA (2021). Investigation of sensory nerve endings in pulvinar, ligamentum teres, and hip joint capsule: A prospective immunohistochemical study of 36 cases with developmental hip dysplasia. Acta Orthop Traumatol Turc.

